# Stimulation of primordial follicle assembly by estradiol-17β requires the action of bone morphogenetic protein-2 (BMP2)

**DOI:** 10.1038/s41598-017-15833-4

**Published:** 2017-11-14

**Authors:** Prabuddha Chakraborty, Shyamal K. Roy

**Affiliations:** 1 Department of Genetics, University of North Carolina at Chapel Hill, Chapel Hill, NC USA; 2 Department of Cellular and Integrative Physiology, and University of Nebraska Medical Center, Omaha, NE USA; 3 Department of Obstetrics and Gynecology, University of Nebraska Medical Center, Omaha, NE USA

## Abstract

Primordial follicle (PF) pool determines the availability of follicles for ovulation in all mammals. Premature depletion of the PF reserve leads to subfertility or infertility. Bone morphogenetic protein 2 (BMP2) promotes PF formation by facilitating oocyte and granulosa cell development. Estradiol-17β (E2) upregulates PF formation in developing hamster ovaries. However, if BMP2 mediates E2 effect is not known. We hypothesize that E2 facilitates the effect of BMP2 on somatic to granulosa cell transition. BMP2 and E2 together significantly upregulated the percentage of PFs in hamster fetal ovaries *in vitro* compared with either of the treatments alone. E2 also promoted BMP2 expression *in vivo*. Inhibition of BMP2 receptors suppressed E2-stimulation of PF formation while knockdown of BMP2 *in vitro* significantly suppressed the E2 effect. In contrast, estrogen receptor blocker did not affect BMP2 action. Inhibition of the activity of E2 or BMP2 receptors, either alone or combined during the last two days of the culture (C6-C8) resulted in a significant decrease in PF formation by C8, suggesting that both BMP2 and E2 action is essential for somatic cell differentiation for PF formation. Together, the results suggest that E2 activates BMP2-BMPR system leading to the formation of primordial follicles.

## Introduction

Successful follicular development is essential for reproduction^1^. During female gametogenesis, oocyte development is supported by surrounding somatic cells^2^. The initial stage of follicle development starts with the breakdown of the oocyte nests concurrent with encapsulation of the oocytes by juxtaposed granulosa cells leading to the formation of primordial follicles (PF) at either fetal or postnatal life in mammals. Primordial follicle endowment in the ovary during development serves as the lifetime reserve, and PFs are constantly recruited into the growing pool to produce ovulatory follicles. It has been suggested that established primordial follicle reserve and dynamic small antral follicle reserve are linked, and a decrease in the size of the established follicle reserve reduces the size of the small antral follicle pool, thus affecting the development of ovulatory follicle and fertility^3^. Depletion of PF reserve can lead to premature ovarian failure with consequent infertility and associated menopausal conditions such as osteoporosis, cardiovascular diseases and cognitive disorders^4^.

During fetal life, appearance and migration of alkaline phosphatase positive primordial germ cells (PGCs) in the genital ridge around E10.5 in the murine ovary marks the first step of ovary development^5^. Rapidly proliferating PGCs undergo incomplete cytokinesis and remain attached by syncytium, thus forming colonies of oogonia, which are called egg nests^6^. After mitosis, oogonia enter the 1^st^ meiotic prophase around E12.5 in mice and E15 in hamsters, and become diplotene stage oocytes by E15 in mice^7^ and postnatal day 3 (P3) in hamsters^8^. Oocytes remain at the diplotene stage throughout the postnatal life until the LH surge induces the resumption of the meiosis. A large number of oogonia undergo apoptosis during their transition into oocytes in mammalian ovaries^9^. Pepling has suggested that during primordial follicle formation in mice, significant oocyte apoptosis coincides with the breakdown of the egg nests^10^, but no such apoptosis has been noted in the hamster ovary during PF formation. However, germ cells apoptosis does occur in the hamster during oogonia to oocyte transition^8^. Following oocyte formation, the undifferentiated ovarian somatic cells abutting the egg nests^11^ differentiate into flattened squamous granulosa cells (GCs) and invade the egg nests. GCs ultimately encapsulate individual oocytes to form PFs^12,13^. The communication between the oocyte and surrounding somatic cells plays an important role in ovarian folliculogenesis^14^.

Several growth factors and hormones have been shown to play important roles in PF formation^15,16^. Emerging evidence suggests that estradiol-17β (E2) at specific concentration, supports early follicular development^17,18^. During gestation, developing fetus is exposed to species-specific levels of E2^19,20^. Higher serum E2 is detected in primate blood during PF formation^21^. Further, reduction of serum E2 by an aromatase inhibitor, Letrozole in baboons leads to decrease in primordial and primary follicle formation in the offspring. Administration of E2 *in vivo* results in increased percentage of PF formation^22^. In mice, despite a fall in serum E2 on the day of birth from the mid-gestation level, serum E2 remains high during PF formation. Further, serum E2 increases 4-fold in newborn mice within 4 hours after birth^23,24^ suggesting that developing ovaries are exposed to E2 throughout the critical period of PF formation. Fetal ovary also expresses aromatase and capable of producing E2 in mice and human^25,26^. In fetal hamsters, aromatase expression is detected in the ovary from E13, and increase in serum E2 is evident in neonatal hamsters before the formation PFs on post-natal day 8 (P8)^18^. Nuclear estrogen receptors are expressed in the developing hamster ovary^27^. Further, exogenously administered E2 promotes PF formation both *in vivo* and *in vitro* in developing hamster ovaries depending on the concentrations^18^. While low concentration supports, higher concentration adversely affects PF formation^18^.

Members of the TGF-β superfamily have been shown to play a crucial role in ovarian folliculogenesis^28^. During early ovary development, bone morphogenetic proteins (BMPs) such as BMP2, BMP4 or BMP8 are required for PGC development^29,30^. Global deletion of BMP2 gene is embryo-lethal in mice, but no information is available about *in vitro* effect of BMP2 on mouse primordial follicle formation. While BMP4 does not affect PF formation in 4-day old rat ovaries in culture, it accelerates primordial to primary follicle transition^17^. It is to be noted that PF formation is already in progress in 4-day rat ovaries. On the other hand, BMP4 has been shown to induce germ cells apoptosis in fetal human ovaries^31^. The effect of BMP2 has not been examined on fetal rat or human ovaries. Therefore, consistent information on BMP2 effect on PF formation across species is not available. The expression of BMP2 is present in developing ovaries during oocyte development and PF formation in the mouse^32^, human^31^ and hamster^8^. BMP2 has been shown to upregulate the meiotic entry of oocytes, and facilitates PF formation in hamsters^8^. BMPs utilize membrane bound bone morphogenetic protein receptors (BMPRs) such as ALK2, ALK3 and ALK6 to mediate their effect. Both ALK3 and ALK6 are expressed in developing hamster ovary before and around the time of PF formation^33^. Other factors like GDF9 have been shown to affect primordial to primary follicle and primary to secondary follicle transition by stimulating granulosa cell proliferation^34–37^. In hamsters, GDF9 promotes PF formation^38^.

Evidence indicates that E2 and BMP2 have mutually supportive role in mammalian cells. E2 has been shown to activate BMP2 promoter activity through either ESR1 or ESR2 in mouse mesenchymal stem cells^39^. It has also been shown that E2 can upregulate BMP2 expression in bone marrow cells, mesenchymal cells and osteogenic cells via ERs^39–41^. Further, BMP2 expression decreases upon ovariectomy in mouse mesenchymal stem cells^42^. While E2 upregulates BMP2 expression in many cell types, BMP2 has been found to facilitate E2 effects as well. BMP2 upregulates the expression of estrogen receptor- α (ESR1) and -β (ESR2) in mesenchymal precursor cells^43^ and, stimulates E2 production in bovine granulosa cells^44^. However, it is not clear how estrogen affects BMP2 expression and BMP2-mediated PF formation during ovary development. Therefore, the goals of this study were to determine if E2 and BMP2 would act cooperatively to induce PF formation or E2 would modulate the effect of BMP2 on PF formation in hamsters. In neonatal hamsters, developing ovary contains scattered egg nests loosely surrounded by somatic cells till postnatal day 7 (P7), and the first cohort of PFs is detected on the postnatal day 8 (P8). The latency of PF formation is also identical *in vivo* and *in vitro*, thus offering an unique window to study PF formation *in vivo* and *in vitro* ^45,46^. We hypothesize that E2 facilitates BMP2 action on the transition of ovarian undifferentiated somatic cells into granulosa cells.

## Results

### E2 and BMP2 act synergistically to stimulate primordial follicle formation

**Figure 1:**
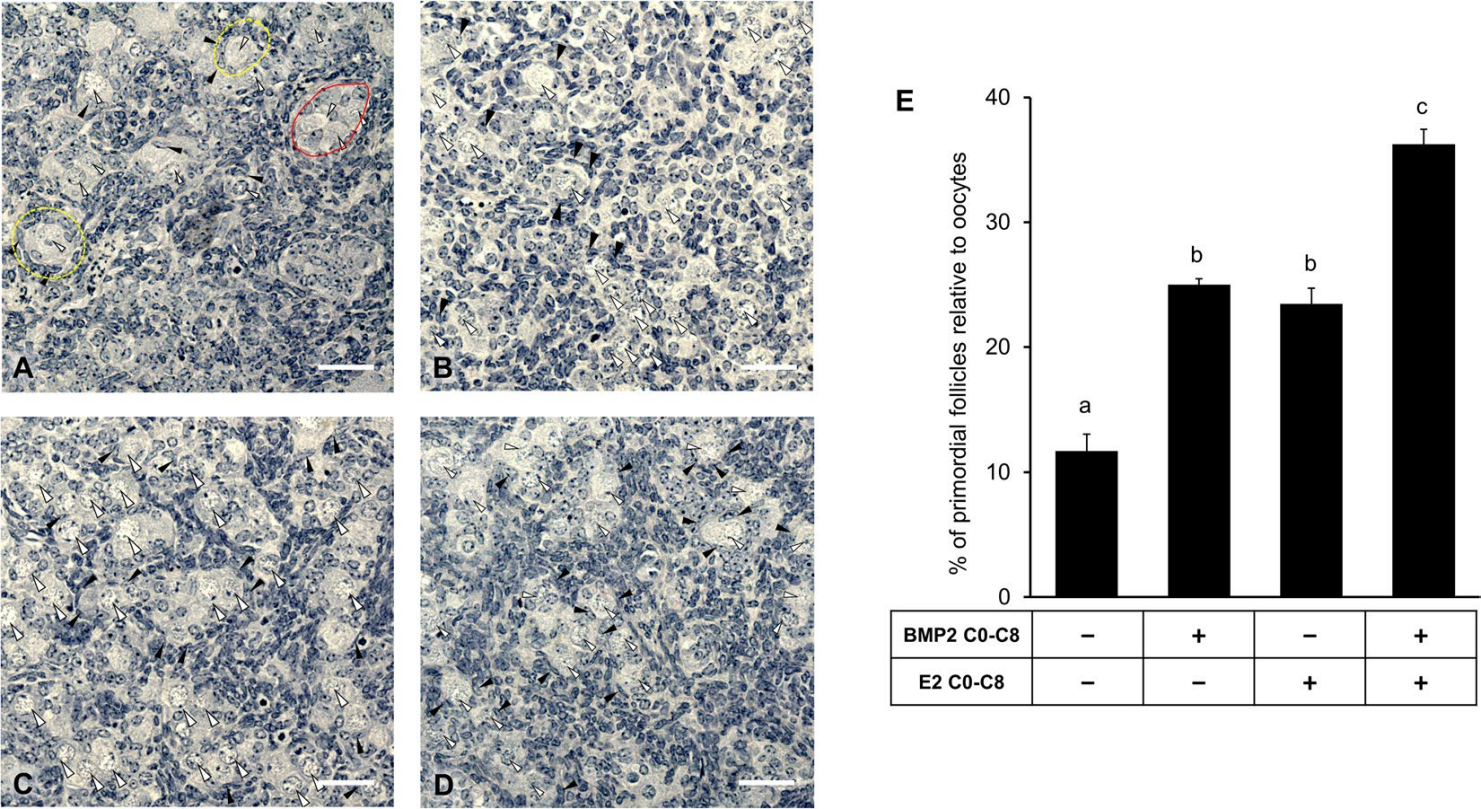
BMP2 and Estradiol-17β (E2) facilitate primordial follicle formation. Primordial follicle index of ovaries treated *in vitro* with either vehicle (**A,E**) or BMP2 (**B**,**E**) or E2 (**C,E**) or BMP2 and E2 in combination (**D,E**) from E15 (C0) through C8. Each bar represents the PF index ± SEM, n = 4. Oocytes are marked with white arrowheads, somatic cells are marked with black arrowheads, oocyte nests are demarcated with a solid red line and primordial follicles are demarcated with yellow broken line. Data were analyzed by one-way ANOVA with Tukey’s post-hoc test. For the bar graph, P < 0.05, bars with different letter, P > 0.05, bars with same letter. For histology images, bar = 50 μm.

To determine whether E2 synergized with BMP2 to promote the assembly of the oocytes and granulosa cells leading to PF formation, ovaries from 15-day old fetuses (E15) were cultured without or with E2, BMP2 or E2 plus BMP2 for 8 days. Morphological analysis revealed that either E2 or BMP2 could significantly increase PF formation compared to untreated ovaries (Fig. 1A–C,E). However, a remarkable synergy in promoting PF formation was noted when ovaries were exposed simultaneously to BMP2 and E2 compared to either of the ligands alone (p < 0.05) (Fig. 1D–E).

### E2 stimulation of PF formation is mediated by BMP2-BMPR system

**Figure 2:**
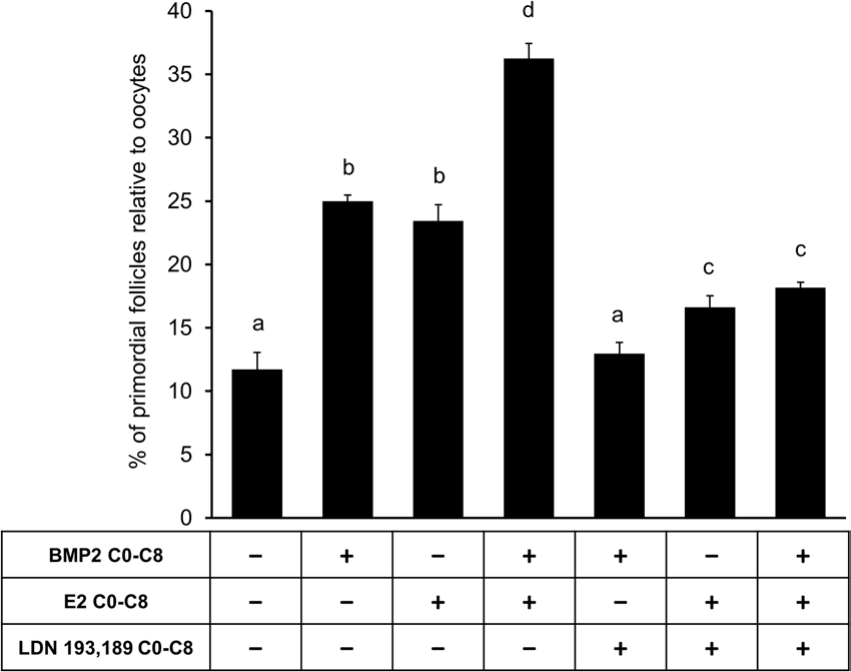
E2 effect on primordial follicle formation is mediated by the activity of ALK2/3. Primordial follicle index after treatment of E15 ovaries from C0 through C8 with either vehicle, BMP2, E2 or E2 plus BMP2 in the presence or absence of ALK2/3 inhibitor LDN 193,189. Each bar represents the PF index ± SEM, n = 4. Data were analyzed by one-way ANOVA with Tukey’s post-hoc test. P < 0.05, bars with different letter. P > 0.05, bars with same letter.

To determine whether E2-stimulated PF formation required BMP2 receptor activation, activities of ALK2 and ALK3 (ALK2/3) were inhibited with LDN193,189, an ALK2/3 inhibitor. Exposure to ALK2/3 inhibitor resulted in a complete (p < 0.05) inhibition of the stimulatory effect of BMP2 on PF formation (Fig. 2). Although, ALK2/3 inhibitor could suppress (p < 0.05) the stimulatory effect of E2 (p < 0.05) or E2 plus BMP2 (Fig. 2) on PF formation, small but significantly higher PF formation was still evident compared to untreated control ovaries (Fig. 2), suggesting that at least part of the E2 effect might be mediated by the activities of BMPR.

**Figure 3:**
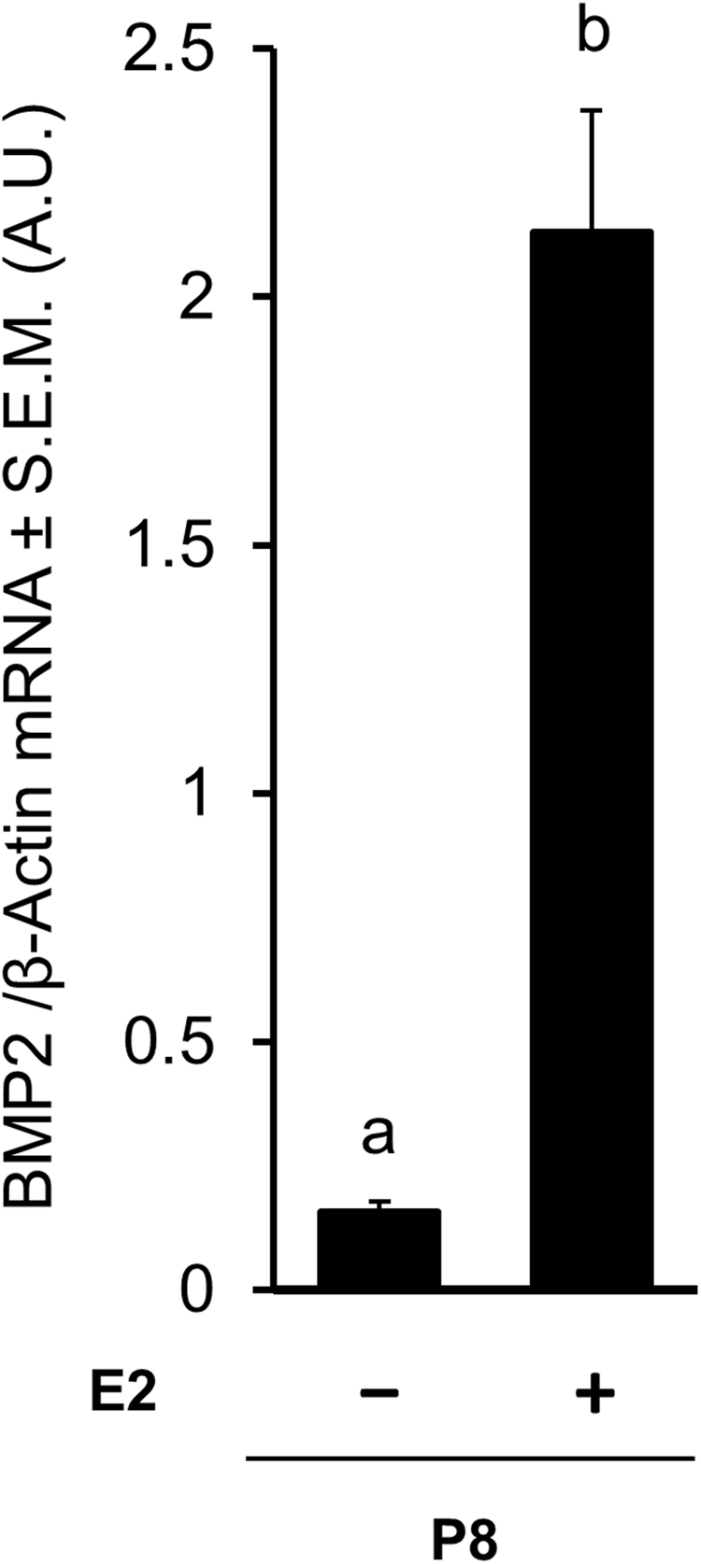
E2 treatment in the hamster ovaries leads to increased level of BMP2 mRNA in postnatal day 8 (P8). RT-qPCR analysis of BMP2 and β-Actin mRNA expression in P8 hamster ovaries following *in vivo*. Each bar represents the ratio of BMP2 and β-actin ± SEM, n = 3. Data were analyzed by unpaired t-test. P < 0.05, bars with different letter.

To verify if E2 would stimulate ovarian BMP2 expression during PF formation, postnatal hamsters were injected without or with E2, and levels of BMP2 mRNA in P8 ovaries was quantified. Appreciable upregulation of BMP2 mRNA expression by P8 was evident for ovaries exposed to E2 (p < 0.05) (Fig. 3).

**Figure 4:**
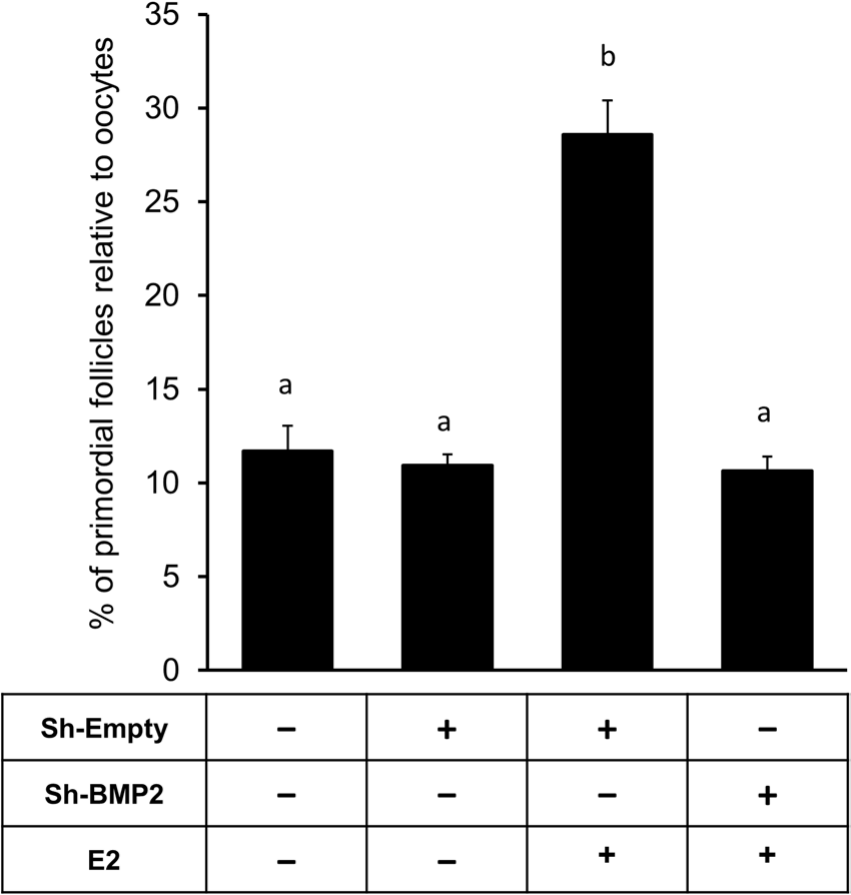
E2-mediated primordial follicle formation requires BMP2. Primordial follicle Index in E15 ovaries cultured from C0 through C8 upon BMP2 knockdown by lentiviral shRNA. Ovaries were cultured with either vehicle or E2 from C2-C8. Each bar represents the PF index ± SEM, n = 4. Data were analyzed by one-way ANOVA with Tukey’s post-hoc test. P < 0.05, bars with different letter. P > 0.05, bars with same letter.

To determine further if BMP2 would act as a downstream effector of E2 in PF formation, ovarian BMP2 expression was knocked down *in vitro* and the effect of E2 on PF formation determined. Morphometric evaluation revealed that whereas empty lentivirus had no effect on either the basal or E2-stimulated PF formation, BMP2 knockdown resulted in a significant reduction in E2-stimulated PF formation (Fig. 4).

### Effect of BMP2 on E2-mediated PF formation *in vitro*

**Figure 5:**
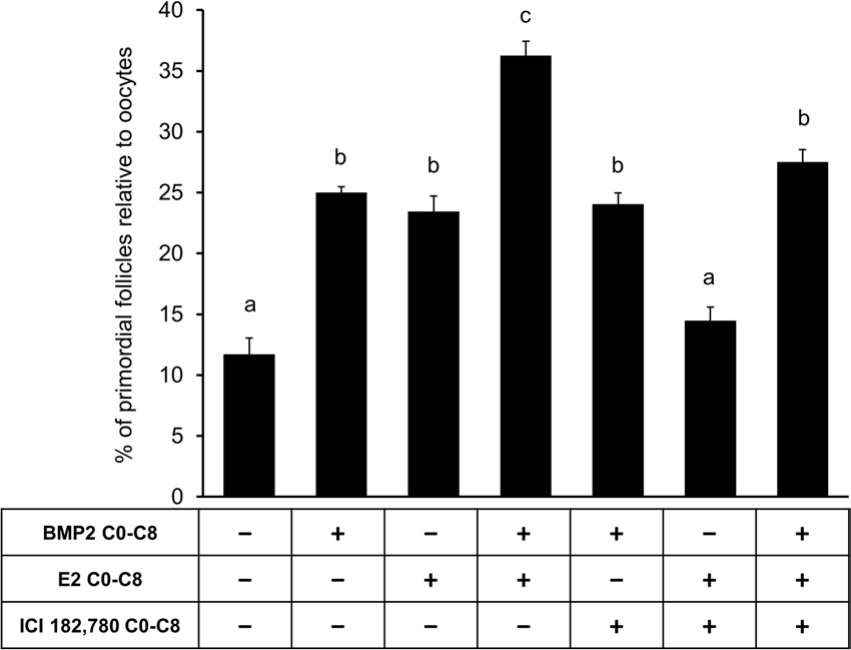
BMP2 effect on primordial follicle formation is independent of the activity of classic estrogen receptor. Primordial follicle index after culture of E15 ovaries with either vehicle or BMP2 or E2 either alone or in combination in the presence or absence of ICI 182,780, an ESR inhibitor from C0-C8. Each bar represents the PF index ± SEM, n = 4. Data were analyzed by one-way ANOVA with Tukey’s post-hoc test. P < 0.05, bars with different letter. P > 0.05, bars with same letter.

To determine if BMP2 promoted PF formation via activation of classic estrogen receptors (ESR), E15 ovaries were cultured for 8 days in the presence of ICI182, 780, an ESR blocker with or without BMP2, E2 or BMP2 plus E2. Whereas complete suppression of E2-stimulated PF formation was evident following ICI182,780 exposure, ESR inhibitor failed to suppress the effect of BMP2 (Fig. 5). On the other hand, ICI182,780 could selectively reduce the effect of E2 when ovaries were cultured with E2 plus BMP2 (Fig. 5), suggesting that E2 might play a major supportive role in BMP2-induced PF formation, but BMP2 effect did not require ESR activities.

### E2 action is required for the differentiation of somatic cells into granulosa cells for PF formation

**Figure 6:**
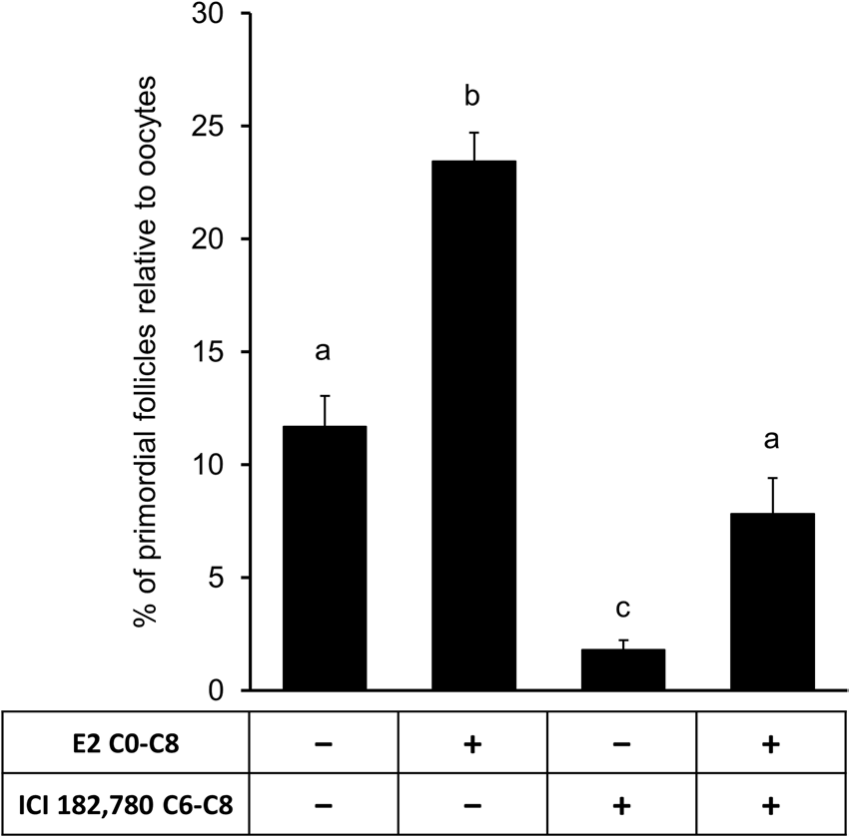
Inhibition of classic estrogen receptors reduces somatic cell differentiation and PF formation. Primordial follicle index in E15 ovaries after culture with either vehicle or E2 from C0-C8 and in the presence or absence of ICI 182,780 from C6-C8. Each bar represents the PF index ± SEM, n = 4. Data were analyzed by one-way ANOVA with Tukey’s post-hoc test. P < 0.05, bars with different letter. P > 0.05, bars with same letter.

To determine if E2 action was required for the differentiation of somatic cells into granulosa cells, E2 binding to estrogen receptor was blocked by ICI182,780 during the last two days, i.e., C6 through C8, of the culture. In hamsters, oogonia to oocyte transition *in vivo* is mostly completed by postnatal day 3, and *in vitro* by C4 upon BMP2 exposure^8^. ICI182,780 remarkably (p < 0.05) suppressed PF formation in control cultures, suggesting the importance of endogenously produced estrogen in basal PF formation (Fig. 6). Exposure of ovaries to the estrogen receptor blocker from C6 through C8 also markedly (p < 0.05) suppressed the stimulatory effect of E2 on PF formation (Fig. 6). The level of suppression was below the basal level, but the values did not reach statistical significance.

### ALK2/3 activities mediate E2-induced somatic cell differentiation leading to PF formation

**Figure 7:**
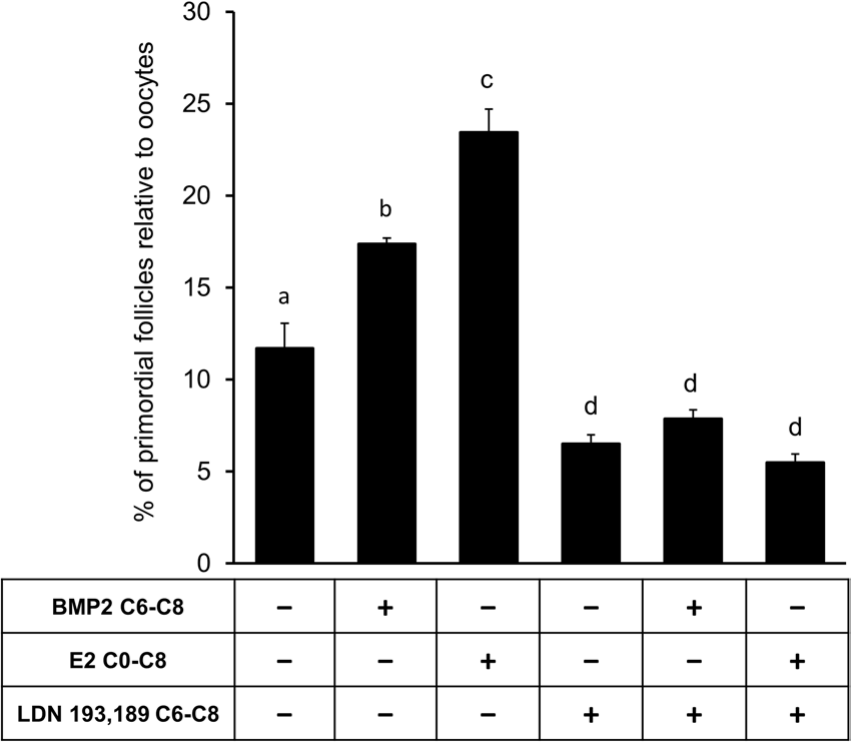
Inhibition of ALK2/3 reduces somatic cell differentiation and PF formation. Primordial follicle index in E15 ovaries after culture with either vehicle or E2 from C0-C8 or BMP2 from C6-C8 in the presence or absence of LDN 193,189, an ALK2/3 inhibitor from C6-C8. Each bar represents the PF index ± SEM, n = 4. Data were analyzed by one-way ANOVA with Tukey’s post-hoc test. P < 0.05, bars with different letter, P > 0.05, bars with same letter.

To determine if E2 utilized BMP2-BMPR system as a downstream effector to promote the differentiation of somatic cells and PF formation, ALK2/3 activity was inhibited during the last two days, i.e., C6 through C8 of the 8-day culture. E15 ovaries were cultured for 8 days in the absence or presence of E2 for 8 days (C0-C8), or BMP2 for C6-C8, or LDN193,189 for C6-C8. In separate 8-day cultures, ovaries were exposed to LDN193,189 plus BMP2 from C6 through C8, or ovaries were cultured with E2 from C0 through C8 and LDN193,189 exposure was limited to C6 through C8. Morphometric analysis revealed that Inhibition of ALK2/3 activity from C6 through C8 significantly (p < 0.05) inhibited the formation of PFs when compared with the vehicle-treated control (Fig. 7); however, BMP2 could not reverse the inhibition (Fig. 7). Notably, exposure to ALK2/3 inhibitor from C6 through C8 before morphologically distinct PFs appear in the ovary markedly blunted the stimulatory effect of E2 on PF formation (p < 0.05) (Fig. 7).

**Figure 8:**
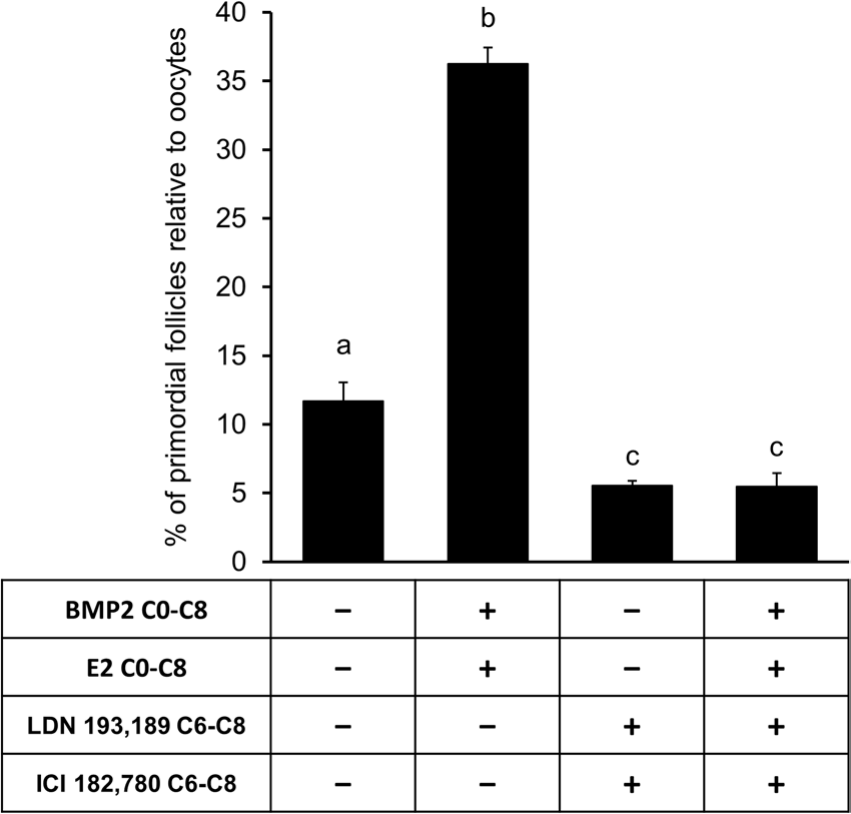
Inhibition of classic estrogen receptors and ALK2/3 together reduces somatic cell differentiation and inhibits PF formation. Primordial follicle index in E15 ovary after culture with either vehicle or BMP2 plus E2 from C0-C8 in the presence or absence of LDN 193,189, an ALK1/2 inhibitor and ICI 182,780, an ESR inhibitor from C6-C8. Each bar represents the PF index ± SEM, n = 4. Data were analyzed by one-way ANOVA with Tukey’s post-hoc test. P < 0.05, bars with different letter, P > 0.05, bars with same letter.

To further determine if simultaneous inhibition of ALK2/3 and ESR activities would interfere with the PF formation, E15 ovaries were cultured for 8 days with vehicle or BMP2 plus E2 from C0 through C8 in the presence or absence of ICI182,780 or LDN193,189. A combined action of BMP2 and E2 remarkably augmented the proportion of PFs, but their effect on PF formation was drastically reduced when the activities of ALK2/3 and ER were abrogated simultaneously (Fig. 8). The combination of inhibitors markedly suppressed the basal PF formation (Fig. 8), thus validating the contribution of exogenously administered ligands.

## Discussion

**Figure 9:**
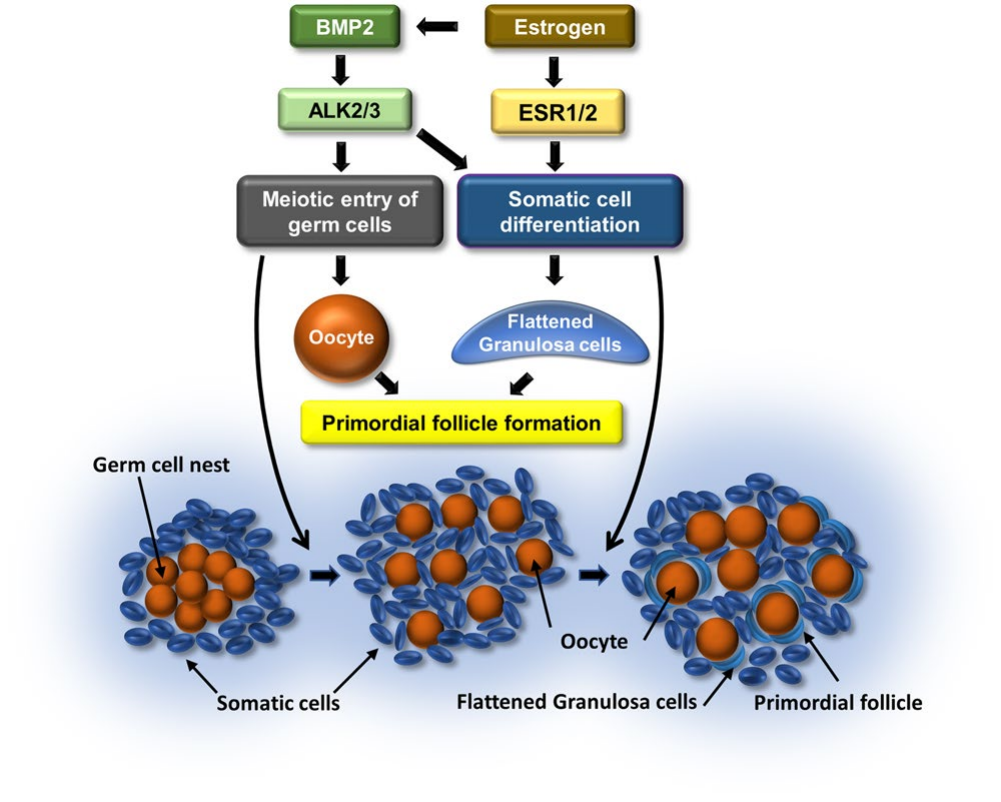
Schematic of the proposed model for E2 and BMP2 interaction facilitating PF formation.

The results of the present study show that either BMP2 or E2 can promote the formation of PFs; however, BMP2 appears to mediate the effect of E2 on the formation of primordial follicles (Fig. 9). The results lends credence to our previous findings^8,18^, and suggest that these two factors act in synergy to regulate morphological changes, which are critical for primordial follicle formation. We have shown earlier that BMP2 affects the transition of oogonia into oocyte during oogenesis^8^, and the transition of somatic cells before the formation of PFs on P8. The upregulation of PF formation in the presence of both E2 and BMP2 in the present study suggests that E2 facilitates BMP2 action perhaps by upregulating BMP2 ligand. Marked increase in ovarian BMP2 mRNA following E2 treatment *in vivo* in the present study supports this hypothesis. On the other hand, suppression of E2-induced PF formation *in vitro* by BMP2 knockdown provides additional evidence to suggest that BMP2 acts downstream of E2 action. Whether E2 potentiates the activities of ALK2/3 is not known. We have not yet detected consistent increase in ALK3 mRNA in postnatal hamster ovaries.

Recent evidence suggests that BMP2 can increase estrogen receptor (ESR) expression in mouse mesenchymal stem cells and promote estrogen production in bovine granulosa cells^43,44^. However, failure of the ESR blocker to suppress BMP2-induced PF formation in the present study suggests that BMP2 action does not engage ESR activities, either by upregulating ovarian E2 or ESR in the fetal hamster ovary. On the other hand, inhibition of ALK2/3 activities by a pharmacological agent leads to the suppression of the effect of BMP2 and E2, either separately or combined on PF formation. ALK2 or ALK3 functions as heterotetramers^47^ and similar to other TGFβ family of ligands, BMP2 has been shown to act via canonical as well as a non-canonical signaling pathways^48^. While the canonical signaling of BMP2 involves SMAD1/5^49,50^, the non-canonical signaling may involve other signaling pathways, such as PI3-kinase/Akt or MAP kinase^48,49^. It has been shown that BMP2 can differentially regulate osteoblast differentiation by temporally activating canonical or non-canonical signaling^51^. We have demonstrated that in the hamster, BMP2 activation of ALK2/3 is essential for PF formation^8^. Whereas BMP2 has been shown to facilitate oocyte formation and somatic cell differentiation during *in vitro* PF formation in the hamster^8^, temporal activation of canonical or non-canonical signaling of BMP2 during PF formation in the hamster requires further investigation. Nevertheless, the results of the present study lead us to postulate that the downstream effect of E2 on fetal ovarian cells requires the participation of BMP2. This contention is further supported by the inhibition of E2-induced PF formation upon BMP2 knockdown.

BMP2 promotes oocyte development in hamster ovaries during 8 days of culture *in vitro* ^8^. Oogonia in the mouse start entering the 1^st^ meiotic prophase by E12.5 and the process is completed by E15^52,53^. In contrast, oogonia in the hamster proliferate as late as E15^8^ and start entering the 1^st^ meiotic prophase on P1, and become diplotene oocytes by P3 *in vivo* ^8^. BMP2 also promotes somatic cell differentiation in hamster ovaries when administered *in vitro* during the last two days (C6-C8) of the organ culture before the appearance of PFs on C8^8^. Marked decrease in basal PF formation by ICI182,780 exposure during the last two days of the organ culture suggests a possible effect of endogenous E2, because the culture medium does not contain phenol red or serum. We have shown that fetal hamster ovary can synthesize E2 *in vitro* ^18^. Further, inhibition of E2-induced PF formation by ICI182,780 exposure from C6 through C8 suggests that E2 promotes differentiation of somatic cells before the formation of PFs. The results of the present study seem to indicate that activation of both BMP2 and E2 receptors is necessary for somatic cell transition during PF formation. Whereas 1 ng/ml (3.67 nM) concentration of E2 *in vitro* stimulates PF formation in the hamster ovary, 10 ng/ml (36.7 nM) concentration causes significant apoptotic changes both in the oocytes and somatic cells^18^. Similarly, reduction of serum E2 by Letrozole in baboons markedly reduces PF formation, but the situation is reversed by exogenously administered E2^22^. Kezele *et al*. have shown that *in vivo* exposure of 4-day old rat ovaries to 10^−6^M E2 markedly upregulates the percentage of PFs, but suppresses PF to primary follicle transition^12^. In contrast, Pepling have shown that daily exposure of newborn mouse ovaries to 10^−7^M (27.24 ng/ml) E2 in culture blocks the breakdown of the egg nests^54^. Similarly, daily *in vivo* administration of 5 mg/kg E2 to newborn mice for four or five days also suppresses the breakdown of the egg nests^54^. These lines of evidence suggest that E2 at very high levels interferes with PF or primary follicle formation in mice or rats. Because E2 is present in nanogram levels in the hamster serum, we have used E2 concentrations to keep the level within the physiological range. Whereas apoptotic germ cells are visible during oocyte development in hamster ovaries *in vitro* ^8^, appreciable oocyte death has not been observed during the formation of PF on P8 (unpublished observation). Failure of BMP2 or E2 to overcome the inhibitory effect of ALK2/3 blocker on PF formation during *in vitro* ovary development from C6 through C8 not only validates the need for BMP2 in somatic cell to flattened granulosa cell transition, but it also suggests that E2 action, at least partly, is mediated by BMP2-ALK2/3 system. Reversal of ICI182,780 inhibition of E2-induced PF formation by BMP2 supports this contention.

Suppression of E2-induced PF formation to the level observed for untreated controls following BMP2 knockdown lends credence to the speculation that endogenous BMP2 maintains basal level of PF formation *in vitro* until BMP2 mRNA declines below the functional level. In contrast to almost instantaneous inhibition of ALK2/3 or estrogen receptor activities, hence, of ligand function by pharmacological receptor blockers, appreciable knockdown of BMP2 mRNA and protein by lentivirus-BMP2 shRNA requires a minimum of 48 h. Therefore, endogenous BMP2 effect is expected to continue until BMP2 mRNA is reduced below the effective level. Therefore, results of the BMP2 knockdown experiment provide additional evidence for the importance of E2 and BMP2 effect on the differentiation of somatic cells into granulosa cells.

In conclusion, the results of this study demonstrate that E2 facilitates the formation of primordial follicles in the hamster ovary. E2 appears to promote the differentiation of somatic cells into granulosa cells, which can assemble with the oocytes to form primordial follicles. At least, part of the E2 effect is mediated by BMP2, which facilitates oocyte formation^8^ and differentiation of somatic cells into granulosa cells (Fig. 9). Whether E2 also affect the transition of oogonia into oocytes remains to be examined.

## Materials and Methods

Recombinant human BMP2 was purchased from R&D Systems (Minneapolis, MN). E2 was purchased from Steraloids, Inc. (Newport, RI). Estradiol 17β cypionate was purchased from Pfizer Company (Kalamazoo, MI). Phenol red-free DMEM, linolenic acid, bovine serum albumin (BSA), and fine chemicals were purchased from Sigma Chemical Company (St. Louis, MO). LDN193,189 was purchased from Sigma Chemical Co. (St. Louis, MO), ICI182,780 was purchased from Tocris Bioscience (Bristol, UK), and human holo-transferrin was obtained from BD pharmaceuticals (San Jose, CA). Plastic embedding medium and supplies were obtained from Electron Microscopy Sciences (Hatfield, Pa). Quantitative RT-PCR primers and probes were synthesized by Sigma Chemical Company. RNeasy mini kit and Taq DNA polymerase were from Qiagen, Inc. (Valencia, CA). All other molecular biology grade chemicals were obtained from Sigma Chemical Company or Fisher Scientific (Pittsburgh, PA).

### Animals and Treatments

Female golden hamsters (*Mesocricetus auratus*) (100–110 g) were obtained from Harlan Laboratories (currently Envigo) and housed in a climate-controlled environment with 14 h light and 10 h dark cycle, and fed ad libitum according to the UNMC Institutional Animal Care and Use Committee and United States Department of Agriculture guidelines. The use of hamsters in the research was approved by UNMC IACUC. All methods were performed in accordance with relevant guidelines and regulations. Females with at least three consecutive estrous cycles were mated in the evening of proestrus, and the presence of sperm in the vagina the next morning was considered day 1 of pregnancy. The gestational period of hamster is 16 days. Pups were born in the afternoon of 16^th^ day of gestation, which was considered the first day of postnatal life. Neonatal hamsters were injected subcutaneously with 1 μg ECP in 20 μl sesame oil at 0900 h on P1 and again on P4. Ovaries from embryos and postnatal pups were collected and flash-frozen in liquid N_2_ for total RNA extraction or used immediately in ovary organ culture as previously described^8,46^.

### Assessment of BMP2 and E2 effect *in vitro*

Ovaries were collected between 0800 and 1000 hours from hamster fetuses on embryonic day 15 (E15) in phenol red-free and serum-free DMEM containing antibiotics at room temperature, cleaned of adherent tissues, and cultured in the presence of modified ITS^+^ (0.1 μg/ml insulin, and 1.25 μg/ml transferrin, 1.25 μg/ml selenium, and 10.7 μg/ml linoleic acid) on a tissue culture insert with 3 µm high density pores as described previously^45,46^, with or without 50ng/ml BMP2 or 1ng/ml E2. Ovaries were pre-treated with 0.5 μM LDN 193,189 (ALK2/3 inhibitor) or 0.1 μM ICI 182, 780 (estrogen receptor (ESR) inhibitor) for 1 hour before adding the ligands as described for specific experiments. Optimum concentrations of ligands and inhibitors were selected based on our prior publications^8,18^, manufacturers’ suggested ED/ID and dose-response analysis. For each group, there were at least three fetal ovaries from different pregnant hamsters. All ovaries were placed in culture by 1300 h of the day of collection, which we considered as culture day 0 (C0) and media was replaced every 48 hours. Ovaries were retrieved at 1200 hours on culture day 8 (C8) and processed for morphometric evaluation of the formation and development of PF. PF formation was evaluated by calculating the number of PFs and oocytes in every 5th section of the entire ovary. Primordial follicle index (percentage of primordial follicles relative to the oocytes) was derived from total number of primordial follicles and oocytes present in each ovary.

### Lentiviral delivery of shRNA construct in ovaries *in vitro*

Nucleic acid sequences to generate BMP2 shRNA (Sense: [phos]TGCAGGTCT TTGCACCAAGATTCAAGAGATCTTGGTCAAAGACCTGCTTTTTTC, Antisense: [phos]TCGAGAAAAAAGCAGGTCTTTGCACCAAGATCTCTTGAATCTTGGTGCAAAGACCTGCA) were designed from hamster BMP2 sequence (accession number: XM_005068676), and the duplex was cloned into pSiCoR vector (Addgene, Cambridge, MA). HEK293FT cells were transfected with the vector containing the inserts and packaging vectors using Fugene HD (Promega, Madison, WI). Media containing lentiviral particles were collected 48 and 72 hours after transfection, purified and concentrated on a sucrose gradient by ultracentrifugation. The pellet was resuspended in Opti-MEM and aliquots stored at −80 °C until use. E15 ovaries were exposed to lentivirus in the presence of 5 ug/ml polybrene for 6 hours and then cultured for 48 h. On culture day 2 (C2), fresh medium containing either vehicle or E2 was added, and ovaries were cultured till C8.

### RNA isolation from ovaries

Ovaries were sonicated in 1 ml Trizol (Invitrogen, Carlsbad, CA), the aqueous phase containing total RNA was separated using chloroform and added to RNeasy mini column (Qiagen, Valencia, CT). RNA was extracted according to the manufacturer’s instructions, and quantified by a NanoDrop 2000 system (Thermo Fisher Scientific, Waltham, MA).

### Reverse transcription and quantitative polymerase chain reaction (RT-qPCR)

Absolute quantitation of a specific mRNA in samples was determined by RT-qPCR. Ovarian total RNA was reverse transcribed using Omniscript cDNA sysnthesis kit (Qiagen, Valencia, CT) according to manufacturer’s protocol. *In vitro* transcription of the partial clone of the gene using AmpliScribe™ T7 High Yield Transcription Kit (Epicenter, Madison, WI) was performed to produce gene-specific mRNA containing the qPCR primer and probe sequences, which was reverse transcribed using Omniscript cDNA sysnthesis kit (Qiagen, Valencia, CT) according to manufacturer’s protocol to produce cDNA for standards. Primers for the qPCR were designed to be internal to the cloned region of the gene specific cDNA. Quantitative PCR was performed using BMP2 specific qPCR primers (F: 5′-GACACCAGGTTAGTGAATCAGAACA-3′, R: 5′-TCTTGGAGACACCTGGCTTCTC-3′), β-Actin-specific qPCR primers (F: 5′-TGACCGAGCGTGGCTACAG-3′, R: 5′-CTTCTCTTTGATGTCACGCACAAT-3′) and gene-specific FAM-labeled Taqman probes (BMP2: 6-FAM-5′-TGCTGTGATGCGGTGGACTGCA-3′-BLACKHOLE, β-Actin: 6-FAM-5′-TCACCACCACAGCCGAGAGGGA-3′-BLACKHOLE), cDNA and HotStar Taq DNA polymerase kit (Qiagen, Valencia, CT) following manufacturer’s protocol in a Chromo 4 thermocycler-detector (MJ research, Canada) and analyzed by Opticon monitor 3 (Biorad, Hercules, CA).

### Statistical analysis

Each group of each experiment was done with three to four ovaries from different pregnant females and each experiment was repeated two to three times. Data were analyzed by either unpaired Student’s t-test or one-way ANOVA with Tukey’s post hoc test for multiple comparisons using GraphPad Prism 5 software (Graph Pad software Inc., La Jolla, CA). The level of significance was P < 0.05.

### Precis

E2 and BMP2 synergistically promote PF formation through classic estrogen receptor and ALK2/3, respectively. Interference of BMP2 production or its receptor function disrupts E2-stimulated PF formation.
